# The Key Role of Complement Receptor CRIg in Kupffer Cell-Mediated Liver Disease Progression

**DOI:** 10.3390/v18040473

**Published:** 2026-04-17

**Authors:** Xin-Zhou Sun, Yan Liu

**Affiliations:** 1 Department of Anaesthesia, First Affiliated Hospital, Zhejiang University School of Medicine, Hangzhou 310003, China; sxz19880429@zju.edu.cn; 2 Department of Blood Transfusion, First Affiliated Hospital, Zhejiang University School of Medicine, Hangzhou 310003, China

**Keywords:** Kupffer cells, CRIg, liver immunity, complement system, immune regulation

## Abstract

Liver diseases, ranging from chronic hepatitis and metabolic dysfunction to cirrhosis and hepatocellular carcinoma, represent a major global public health burden. As an immune-privileged organ, the liver harbors a unique and intricate immune microenvironment, which plays a dual role in pathogen clearance and chronicity. Kupffer cells (KCs), the primary resident macrophages in the liver, constitute the first line of defense in liver innate immunity and play complex and important roles in pathogen recognition, phagocytosis, and the regulation of liver inflammation and immune responses. The complement receptor of the immunoglobulin superfamily (CRIg) is a membrane receptor that is specifically expressed on KCs. It serves not only as a sentinel for the liver against pathogen invasion but also as a sophisticated regulator for maintaining immune homeostasis. As a key component of the liver’s immune system, CRIg can efficiently mediate the clearance of complement-opsonized particles, thereby playing multidimensional roles in pathogen clearance, antigen cross-presentation, and the establishment of immune tolerance, functioning as both a “pathogen catcher” and an “immune brake.” This review focuses on the CRIg molecule, detailing its mechanisms in the recognition and phagocytic clearance by KCs, and its subsequent impact on hepatic immune responses. Furthermore, we explored the potential involvement of CRIg in the pathological progression of diverse liver diseases, including persistent inflammation, fibrosis, and hepatocarcinogenesis. This synthesis provides novel insights into the immunopathology of liver diseases and establishes a theoretical foundation for developing CRIg-targeted therapeutic strategies.

## 1. Introduction

Liver diseases, ranging from persistent hepatitis to cirrhosis and hepatocellular carcinoma (HCC), represent a formidable challenge to global public health. According to World Health Organization estimates, viral hepatitis (particularly hepatitis B virus, HBV) remains a significant cause of liver-related morbidity, affecting approximately 257 million people globally [1,2], alongside other increasingly prevalent etiologies such as alcohol-related liver disease [3]. The progression of liver disease is a dynamic pathological process influenced by complex interactions between injurious agents and host immune responses. The liver serves not only as the primary metabolic center but also as a unique immune organ, possessing abundant innate immune cells and a specialized immune microenvironment that plays a decisive role in determining the balance between homeostasis, injury resolution, or progression to chronic pathology [4]. The pathological process of liver disease involves complex host immune responses, in which the liver’s innate immune system plays a central role in the balance between immune tolerance and inflammatory injury, directly influencing the direction of disease progression.

The inherent immune tolerance of the liver plays a dual role in response to pathogenic insults: it must initiate effective innate immune responses to control injury while suppressing excessive inflammation to maintain tissue homeostasis. Kupffer cells (KCs) are resident macrophages within liver sinusoids, accounting for 80–90% of tissue macrophages in the body and comprising 20–35% of all non-parenchymal cells in the liver [5]. Under physiological homeostasis, these cells, which are derived from embryonic progenitors, maintain an immunotolerant phenotype. However, they are simultaneously primed to detect danger signals by utilizing a broad array of pattern recognition receptors (PRRs), such as Toll-like receptors (TLRs), to sense pathogen-associated molecular patterns (PAMPs) and damage-associated molecular patterns (DAMPs) [6,7]. As professional antigen-presenting cells (pAPCs), KCs process and present antigens to orchestrate the adaptive immune response, recruiting T cells and other effectors via the secretion of a spectrum of cytokines and chemokines, thereby modulating hepatic inflammation and fibrogenesis [8,9]. Importantly, KCs exhibit remarkable functional plasticity in response to injury, rather than adhering to a singular proinflammatory trajectory. Their functional state (such as classical M1 or alternative M2 polarization) is intricately shaped by the local hepatic microenvironment and dynamic interactions with hepatocytes, hepatic stellate cells, and liver sinusoidal endothelial cells [10].

Among the key molecular regulators expressed by KCs, the complement receptor of the immunoglobulin superfamily (CRIg) has emerged as a central hub bridging complement-mediated opsonization to pathogen clearance and immune regulation [11]. Functioning as a transmembrane protein predominantly localized on tissue-resident macrophages, particularly liver KCs, CRIg specifically recognizes and binds complement fragments C3b and iC3b to facilitate the phagocytic clearance of complement-opsonized pathogens and particles [12], and plays important roles in various immune-mediated diseases. Beyond this classical phagocytic role, CRIg acts as a member of the B7 protein superfamily and functions as a T-cell co-inhibitory ligand. By directly engaging its putative receptor on T cells, CRIg effectively suppresses T-cell activation and proliferation, representing a pivotal mechanism for inducing immunological tolerance [13]. Through these dual functions, CRIg plays a critical role in various immune-mediated diseases. The pathophysiological significance of CRIg is particularly evident in metabolic liver diseases; for instance, the pronounced downregulation of CRIg in high-fat diet (HFD)-induced NAFLD compromises its immune inhibitory capacity, drives M1 macrophage polarization, and subsequently activates NF-κB signaling, thereby exacerbating hepatic inflammation [14,15,16]. In addition to metabolic dysfunction, recent studies have identified CRIg as a crucial immune checkpoint molecule in hepatocellular carcinoma (HCC), where its blockade can enhance anti-tumor T-cell cytotoxicity [17]. Concurrently, CRIg-mediated clearance of gut-derived microbial products from the portal circulation provides essential protection against both metabolic and alcoholic liver disease [15,18]. Consequently, by integrating robust phagocytic clearance with potent immunosuppressive capabilities, CRIg acts as a unique bifunctional molecule that profoundly dictates the progression and outcome of diverse liver diseases.

Given the central role of Kupffer cells in the hepatic immune microenvironment and disease progression, coupled with the distinctive expression and pivotal functions of CRIg in this cell population, this review systematically dissects the multifaceted roles of CRIg across liver disease progression. Our focus was specifically on the CRIg-expressing KC subset, examining its mechanistic contributions to a spectrum of critical immunological and pathological processes, including pathogen clearance, induction of immune tolerance, modulation of hepatic inflammation, progression of fibrosis, and pathogenesis of HCC. In addition, we critically evaluated the therapeutic potential and inherent challenges of targeting CRIg as a novel immunotherapeutic strategy for intervening in chronic liver disease progression. By synthesizing and critically appraising contemporary research, this review aims to provide novel insights into the mechanisms of immune pathogenesis of liver diseases and establish a robust conceptual framework for the development of innovative, CRIg-targeted therapeutic interventions.

## 2. Kupffer Cells: Sentinels and Key Immune Regulators of the Liver

**Figure 1:**
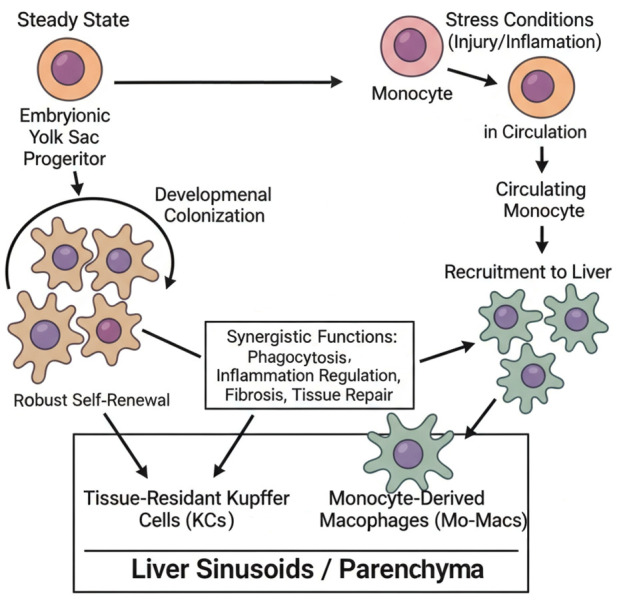
Ontogeny and maintenance of Kupffer cells (KCs). Schematic illustrating the dual origin of hepatic macrophages. Under homeostatic conditions, the liver-resident Kupffer cell (KC) pool is predominantly established and maintained by self-renewing progenitors originating from the embryonic yolk sac (solid arrows). In contrast, during inflammatory stress or liver injury, circulating bone marrow-derived monocytes are recruited to the liver parenchyma and differentiate into monocyte-derived macrophages (Mo-Macs) (dashed arrows). Both populations act in concert within the hepatic sinusoids to mediate phagocytosis, immune regulation, and tissue repair.

Kupffer cells (KCs), strategically positioned within the hepatic sinusoids, are the predominant population of tissue-resident macrophages and serve as the cornerstone of the liver’s innate immune system. They act as essential “sentinels” and “scavengers” by phagocytosing pathogens, endotoxins, apoptotic cells, senescent erythrocytes, and immune complexes from the circulation, thereby preserving hepatic and systemic homeostasis [19,20]. The long-standing paradigm previously posits that KCs are primarily derived from bone marrow-derived monocytes that migrate to the liver. However, this dogma has been fundamentally overturned by lineage-tracing technologies, which have unequivocally demonstrated that under homeostatic conditions, the KC pool is predominantly seeded by progenitors originating from the embryonic yolk sac [6]. These embryonic progenitors colonize the fetal liver and sustain their population throughout adulthood through robust self-renewal. In contrast, under stress conditions such as liver injury or inflammation, circulating bone marrow-derived monocytes are extensively recruited to the liver parenchyma [21]. These monocytes subsequently differentiate into monocyte-derived macrophages, which cooperate with resident KCs to orchestrate key processes, including modulation of inflammatory responses, progression of fibrosis, and promotion of tissue repair [22]. The detailed procedure for KCs lineage tracing is presented in Figure 1.

**Figure 2:**
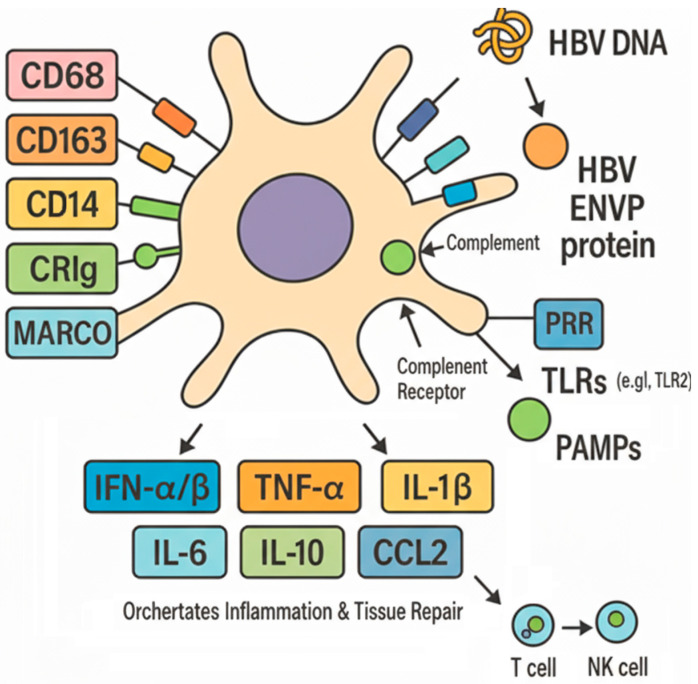
Functional characteristics of human Kupffer cells (KCs). Human Kupffer cells express a repertoire of characteristic surface markers (e.g., CD68, CD163, CRIg) and diverse pattern recognition receptors (PRRs), including Toll-like receptors (TLRs). Through these receptors, KCs sense pathogen-associated molecular patterns (PAMPs) and damage-associated molecular patterns (DAMPs). Upon activation, KCs secrete a broad spectrum of cytokines and chemokines, including pro-inflammatory factors (TNF-α, IL-1β, IL-6, type I interferons), anti-inflammatory mediators (IL-10), and chemokines (e.g., CCL2). This secretory profile critically shapes the hepatic immune microenvironment, influencing both immune defense and immunopathology.

Recent advances in understanding KC homeostasis have identified key molecules essential for KC maintenance. Zhao et al. demonstrated that ALK1 (activin receptor-like kinase 1) signaling is required for KC homeostasis and the prevention of liver fibrosis, with ALK1 deficiency leading to KC depletion, increased susceptibility to bacterial infection, and exacerbated fibrogenesis [23], which has been shown to be critical for sustaining the KC identity and self-renewal capacity. Furthermore, the advent of high-throughput technologies, most notably single-cell RNA sequencing, has revolutionized our understanding of KC biology by unveiling their profound functional and phenotypic heterogeneity in both physiological and pathological states [24,25]. This heterogeneity enables the classification of KCs into distinct subpopulations, defined by unique transcriptional profiles and surface marker expression, with each subset executing specialized functions in hepatic immune surveillance, metabolic regulation, and disease pathogenesis [26]. In mice, resident KCs are predominantly identified by the expression of F4/80, CD68, Tim4, CRIg/Vsig4, Clec2, and Clec4f, whereas human KCs are defined by a combination of markers including CD68, CD163, CD14, CRIg, and MARCO [27,28]. Functionally, KCs express diverse pattern recognition receptors (PRRs) [29,30,31] and complement receptors. Upon activation by these ligands, KCs orchestrate the hepatic immune milieu by secreting a broad spectrum of cytokines and chemokines, including type I interferons (IFN-α/β), TNF-α, IL-1β, IL-6, IL-10, and IL-12 [32,33,34,35,36]. These mediators play pivotal regulatory roles in recruiting and activating immune cells (e.g., T cells and NK cells), thereby orchestrating inflammatory responses and tissue repair within the liver. The surface markers and pro-inflammatory factors of the KCs are shown in Figure 2.

Within the context of liver diseases, KCs exhibit profound functional duality, serving as both defenders against pathogens and potential contributors to tissue damage. Studies have demonstrated that KCs serve as a critical frontline defense against diverse pathogens. For instance, KCs efficiently capture and clear circulating bacteria through complement receptor-mediated phagocytosis. Zeng et al. demonstrated that CRIg functions as a macrophage pattern recognition receptor to directly bind and capture blood-borne Gram-positive bacteria [37] while a dual-track clearance mechanism involving both CRIg-expressing KCs and platelets balances rapid blood sterilization with immune adherence to facilitate adaptive immunity [38]. Furthermore, KC-mediated fungal clearance through blood filtration effectively limits fungal dissemination [39], and the STING signaling pathway within KCs has been identified as a critical antiviral defense mechanism across multiple hepatic viral infections. Recent work by Wang et al. has shown that in severe alcohol-associated cirrhosis, infiltrating monocyte-derived macrophages progressively replace resident KCs, with these infiltrating macrophages playing diverse and sometimes detrimental roles in disease progression, underscoring the critical necessity of preserving the resident CRIg+ KC pool [40].

Conversely, KCs can also contribute to immunopathology and disease progression under pathological conditions. In metabolic liver disease, KC activation by gut-derived endotoxin (LPS) translocating via a compromised intestinal barrier drives hepatic inflammation through TLR4/NF-κB signaling, promoting steatohepatitis progression [41]. In alcoholic liver disease, KC-derived pro-inflammatory cytokines and chemokines (TNF-α, IL-1β, CCL2) directly inflict hepatocyte injury and recruit neutrophils. Simultaneously, KC-secreted TGF-β potently activates hepatic stellate cells (HSCs), triggering a fibrogenic cascade characterized by excessive extracellular matrix deposition that culminates in liver fibrosis and cirrhosis [42]. Furthermore, in systemic inflammatory states such as sepsis, Sun et al. identified a *YTHDF1*/*KLF2*/*VSIG4* axis that regulates KC polarization toward an anti-inflammatory phenotype, revealing a novel mechanistic pathway by which CRIg expression modulates KC function to prevent excessive immunopathology [43]. These findings collectively highlight the dual nature of KCs as both guardians of hepatic homeostasis and contributors to disease progression, dictated by the specific microenvironmental context.

## 3. CRIg: A Key Complement Receptor on Kupffer Cells

### 3.1. Overview of CRIg Molecular Structure

CRIg (Complement receptor of the immunoglobulin superfamily), also known as V-set and immunoglobulin domain containing 4 (*VSIG4*) or Z39Ig, was first identified by Helmy et al. in 2006 as a prominent surface protein on both murine and human Kupffer cells [12]. As a type I transmembrane glycoprotein belonging to the immunoglobulin superfamily (IgSF), CRIg was initially categorized as a B7-related protein due to structural homology in its extracellular domain, though it possesses distinct functional attributes. The gene encoding the human CRIg is located on chromosome Xq11.1, with its murine ortholog situated in the syntenic region A1.1. Human and mouse CRIg proteins exhibit high amino acid sequence homology (~70%) with particularly strict conservation within the IgV domain responsible for ligand recognition. This evolutionary conservation strongly implies a universally conserved function as a key complement receptor in mammalian species [44].

**Figure 3:**
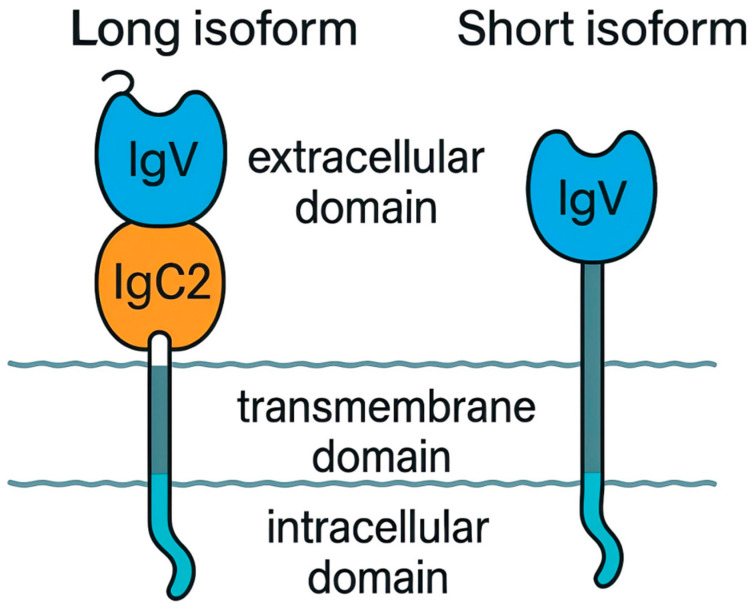
Structural isoforms of the human complement receptor CRIg (*VSIG4*). Diagram depicting the two major alternatively spliced isoforms of the human CRIg transmembrane protein. The long isoform (CRIg-L) possesses an extracellular domain composed of an N-terminal immunoglobulin V-type (IgV) domain, responsible for high-affinity ligand binding, and a C-terminal immunoglobulin C2-type (IgC2) domain. The short isoform (CRIg-S) contains only the IgV domain. Both isoforms are specifically and highly expressed on Kupffer cells, where they mediate phagocytosis of complement-opsonized particles and immunoregulatory functions.

Structurally, the mature CRIg protein comprises three functional domains: an extracellular domain (ECD), transmembrane domain (TMD), and intracellular domain (ICD). The ECD serves as the primary functional module, directly mediating ligand binding and downstream biological effects. In humans, CRIg is alternatively spliced into two major isoforms, a long form (CRIg-L) and a short form (CRIg-S). The canonical CRIg-L features an ECD composed of an N-terminal V-type immunoglobulin (IgV) domain followed by a C2-type immunoglobulin (IgC2) domain [44] whereas CRIg-S retains only the IgV domain. Within this architecture, the IgV domain acts as the critical functional unit, conferring high-affinity binding to the complement fragments C3b and iC3b. While the exact role of the IgC2 domain remains elusive, it is hypothesized to contribute to protein stability, facilitate dimerization, or mediate interactions with other membrane proteins [45]. The TMD anchors CRIg within the plasma membrane, whereas the short ICD lacks canonical immunoreceptor tyrosine-based activation (ITAM) or inhibitory (ITIM) motifs. The major isoforms of human CRIg are shown in Figure 3.

### 3.2. Expression Profile and Regulation of CRIg

A hallmark of CRIg is its highly restricted tissue- and cell-specific expression profile, which is remarkably conserved between the murine and human systems. High-level expression is predominantly confined to select populations of tissue-resident macrophages, most notably liver Kupffer cells, peritoneal macrophages, and alveolar macrophages [46]. In contrast, CRIg expression levels are very low or undetectable in the vast majority of immune cells (e.g., monocytes, lymphocytes, dendritic cells) and other non-macrophage cell types [12,13,47]. This restricted expression pattern strongly implies an indispensable role for CRIg in the specialized functions executed by these macrophage populations, particularly in maintaining local tissue homeostasis and ensuring the efficient clearance of circulating debris, including pathogens, apoptotic debris, and immune complexes [13]. In the liver, the vast majority of mature tissue-resident KCs stably express high levels of CRIg [48], establishing it as a definitive surface marker to discriminate resident KCs from infiltrating monocyte-derived macrophages during inflammatory insults. Furthermore, CRIg expression is not static but dynamically regulated in response to pathological stimuli. Perveen et al. reported that during sepsis, CRIg expression on monocytes and macrophages is significantly upregulated during acute infection but downregulated in chronic stages, suggesting its utility as a dynamic biomarker for immune status in liver disease [49].

At the cellular level, the expression of CRIg in KCs is highly plastic and aligns with macrophage polarization. Consistently, in vitro polarization models reveal an enrichment of CRIg in M2 (anti-inflammatory) macrophages relative to M1 (pro-inflammatory) phenotypes [50]. This expression bias aligns seamlessly with CRIg’s role in maintaining hepatic immune tolerance, promoting tissue repair, and dampening excessive inflammatory responses. Clinically, CRIg expression on KCs is profoundly altered across a spectrum of acute and chronic liver diseases, such as viral hepatitis, non-alcoholic fatty liver disease (NAFLD), liver fibrosis, and hepatocellular carcinoma. These alterations frequently correlate with disease severity and progression, suggesting that CRIg may represent a potential biomarker and viable therapeutic target [14,51].

## 4. Functions of CRIg in Liver Immunity

### 4.1. Clearance of Pathogens and Circulating Particles via CRIg

As a pivotal phagocytic receptor on KCs, CRIg recognizes and binds pathogens primarily through two distinct but complementary mechanisms: complement C3b/iC3b-mediated recognition and direct recognition of pathogen surface molecules. In complement-dependent recognition, CRIg binds to complement C3b/iC3b [12], which are common end products of both the classical and alternative complement pathways. When pathogens are targeted by the complement system and become opsonized, CRIg-expressing KCs can efficiently recognize and engulf these C3-coated particles. This capacity positions CRIg as a central mediator of KC-driven clearance of systemic pathogens. The pioneering work by Helmy et al. established that CRIg’s extracellular IgV domain mediates this specific, high-affinity interaction [12]. Indeed, CRIg-mediated phagocytosis has been shown to be particularly efficient in clearing particles opsonized with low densities of C3b/iC3b, a scenario where other classical complement receptors may be less effective [12]. This highly efficient phagocytic function is critical for blood sterilization and the removal of circulating apoptotic cells and immune complexes. The role of CRIg in mediating KC binding to complement-coated bacteria, fungi, and adenoviruses has been well-documented [12,52,53]. Beyond opsonization, CRIg directly recognizes pathogen-associated molecular patterns. In a landmark study, Zeng et al. demonstrated that CRIg functions as a bona fide pattern recognition receptor (PRR) for Gram-positive bacteria by directly binding lipoteichoic acid (LTA) on Staphylococcus aureus, facilitating the capture of blood-borne pathogens independently of complement opsonization [37]. However, this mode of recognition appears to be primarily restricted to Gram-positive bacteria, and whether this direct binding extends to viral pathogens remains unclear.

Beyond bacterial clearance, CRIg plays a critical role in removing gut-derived microbial products from the portal circulation. Luo et al. revealed that CRIg+ macrophages capture gut microbiota-derived extracellular vesicles (mEVs) from the bloodstream via a C3-dependent opsonization mechanism, preventing the accumulation of microbial DNA in hepatocytes and hepatic stellate cells, thereby inhibiting the activation of the cGAS/STING pathway and subsequent fibrogenic inflammation [18]. This mechanism has been further validated in inflammatory bowel disease (IBD), where CRIg+ macrophage deficiency was shown to enhance inflammation due to impaired clearance of gut microbiota-derived extracellular vesicles [54]. These findings establish CRIg-mediated clearance as a critical barrier function that protects the liver from gut-derived inflammatory insults.

### 4.2. CRIg Regulation of Kupffer Cell Functional State and Cytokine Secretion

Beyond its canonical role in phagocytosis, an expanding body of evidence has highlighted the function of CRIg as a key modulator of KC activation and cytokine production. Specifically, CRIg signaling often serves to impose a negative feedback loop, thereby inhibiting macrophage activation and pro-inflammatory cytokine production, which is of particular relevance across diverse liver disease contexts. Under homeostatic conditions, high CRIg expression in KCs is thought to contribute to their quiescent state, thus preserving hepatic immune tolerance [55]. During inflammatory challenges, it is hypothesized that CRIg expression levels modulate the responsiveness of KCs to PAMPs [19]. For example, some studies in vitro or in animal models have found that CRIg expression levels are negatively correlated with macrophage secretion of pro-inflammatory cytokines such as TNF-α and IL-6 [56,57], but positively correlated with anti-inflammatory cytokines such as IL-10, suggesting that CRIg favors the establishment of an immunosuppressive environment [58]. The precise molecular mechanisms underlying CRIg-mediated immunosuppression have not been fully elucidated, but several non-mutually exclusive pathways have been proposed. (1) Upon ligand binding, CRIg may transduce an inhibitory signal. Although lacking a classical ITIM, its intracellular domain could contain ITIM-like motifs that recruit phosphatases such as SHP-1/SHP-2, which in turn dephosphorylate key adaptors (e.g., IRAKs and TRAF6) in downstream TLR signaling, effectively dampening NF-κB activation. (2) Regulating receptor complexes: CRIg may form functional complexes with other membrane proteins, such as TLRs, through lateral interactions (cis-interaction), directly affecting the signal transmission efficiency of the latter. (3) Influencing macrophage polarization: By inhibiting M1-type related signaling pathways and promoting IL-10 secretion, CRIg powerfully drives KC polarization toward the M2-type (anti-inflammatory/tissue repair) [59]. A deeper mechanistic dissection of these mechanisms can provide key targets and novel approaches for developing immunotherapies targeting CRIg to intervene in the progression of liver diseases.

### 4.3. CRIg Effects on Liver Immune Responses and T Cell Function

In addition to its cell-intrinsic regulatory effects on KCs, CRIg functions as a B7/CD28 superfamily co-inhibitory ligand, enabling it to extrinsically modulate the activity of other immune cells, most notably T cells, through direct cell–cell contact [58,59]. The strategic positioning of CRIg-expressing KCs within the liver sinusoids, where they are in close proximity to trafficking T cells, provides an anatomical substrate for this critical immunoregulatory crosstalk [60]. Extensive experiments have confirmed that CRIg is an effective negative regulator of T cell responses, capable of directly inhibiting the activation and proliferation of mouse and human T cells [47]. Mechanistically, CRIg can effectively inhibit early signaling pathways of T cell activation, such as the ERK-MAPK and Akt-mTOR pathways, thereby suppressing T cell proliferation and cytokine production [13]. CRIg may exert its inhibitory effects by binding to unknown receptors on T cells, broadly attenuating TCR signaling cascades [61]. In vivo, administration of CRIg-Ig fusion proteins effectively suppress the induction of cytotoxic T lymphocytes (CTLs) and T helper-dependent humoral responses. Recent work by Ma et al. deepens our understanding of CRIg’s T cell-suppressive functions. They showed that *VSIG4+* tissue-resident macrophages silence T cells within the tumor microenvironment via a “dual suppressive” mechanism involving IL-11 secretion and *VSIG4*-mediated co-inhibition [62,63], establishing CRIg as an active immunosuppressive hub. This broad regulatory scope is further supported by Pan et al., who found that *VSIG4+* tumor-associated macrophages drive neutrophil infiltration and impair CD8+ T cell function [64]. Beyond oncology, CRIg is essential for hepatic immune homeostasis; CRIg-deficient mice fail to induce tolerance in hepatic T and NKT cells [65]. Intriguingly, in the context of severe inflammation, forced CRIg expression protects against MHV-3-induced fulminant hepatitis by reprogramming macrophage mitochondrial pyruvate metabolism to curb their activation. Together, these studies highlight CRIg as a multifaceted, context-dependent immune regulator, making it a compelling target for liver disease therapeutics.

## 5. The Role of CRIg in the Progression of Liver Diseases

Liver diseases encompass a spectrum of pathological conditions, including viral hepatitis, non-alcoholic fatty liver disease (NAFLD), alcoholic liver disease (ALD), autoimmune hepatitis, and hepatocellular carcinoma (HCC). Kupffer cells (KCs) are central regulators of the hepatic immune microenvironment and are critically involved at every stage of this pathogenic cascade [5,66]. Consequently, the functional state of CRIg molecules highly expressed in KCs is likely to profoundly influence the trajectory of liver diseases.

### 5.1. CRIg and Hepatitis B Virus Infection

Hepatitis B virus (HBV) is considered to have limited direct cytopathic effects; rather, chronic hepatitis B (CHB) progression is predominantly controlled by the host immune response. This unique pathogenesis renders HBV infection a paradigmatic model for studying how CRIg-mediated KC functions shape liver disease trajectories. Indeed, dysregulation of macrophage subsets is a hallmark of CHB pathogenesis. In the context of HBV infection, CRIg expression and function are of particular interest. Guo et al. reported that the CRIg homolog Z39Ig is downregulated on macrophages by IFN-γ in patients with chronic HBV infection, suggesting that the inflammatory milieu of CHB may compromise CRIg’s protective immunoregulatory functions [67]. Consistent with this finding, a single-cell transcriptomic study by Tang et al. identified *VSIG4* as a novel biomarker of immune metabolic disruption in HBV-related acute-on-chronic liver failure (HBV-ACLF), with *VSIG4* expression significantly altered in hepatic macrophage populations during disease progression [68]. This metabolic disruption is closely linked to dysfunctional immune responses, as recently demonstrated. Clinically, low *VSIG4* expression has been associated with poor prognosis in hepatocellular carcinoma patients with hepatitis B infection [53]. This observation, combined with the newly demonstrated role of *VSIG4* in suppressing tumor-specific T cell immunity in HCC [17], suggests that CRIg downregulation during chronic HBV infection may dismantle an important brake on hepatic immune activation, potentially contributing to both immune-mediated liver damage and impaired tumor surveillance. Expanding on this clinical relevance, a recent single-cell study of peripheral immune responses in chronic HBV patients further revealed altered *VSIG4* expression patterns in monocyte and macrophage populations, correlating with disease stage and treatment response [69].

During HBV infection, KCs play dual roles: they execute antiviral clearance functions and simultaneously mediate immune tolerance that facilitates viral persistence. CRIg is likely involved in both aspects—its phagocytic capacity may contribute to the clearance of complement-opsonized viral particles and immune complexes, while its potent T cell inhibitory function may promote the functional exhaustion of HBV-specific T cells. However, direct evidence demonstrating that CRIg on KCs specifically inhibits HBV-specific T cells in vivo remains limited, and further studies are needed to delineate CRIg’s precise role in the immunopathogenesis of CHB.

Interestingly, multiple retrospective clinical studies have reported a significantly reduced incidence of liver metastases from colorectal cancer and other gastrointestinal adenocarcinomas in patients with previous or concurrent hepatitis B virus (HBV) infection. A landmark study of 4033 newly diagnosed colorectal cancer patients demonstrated that concomitant HBsAg positivity was associated with a significantly lower risk of synchronous colorectal liver metastasis (CRLM) [70]. This protective effect was further corroborated by a study of 3132 patients with 5-year follow-up, showing that HBV infection status differentially impacts synchronous and metachronous liver metastasis [71]. While the mechanisms underlying this phenomenon remain to be fully elucidated, it is hypothesized that HBV-induced immune remodeling of the hepatic microenvironment may enhance immune surveillance and clearance of circulating tumor cells, potentially involving CRIg-expressing Kupffer cells. However, direct evidence linking CRIg to this protective effect is currently lacking, and further studies are needed to test this hypothesis and determine whether these findings extend to other digestive tract adenocarcinomas.

### 5.2. CRIg and Hepatic Fibrosis

Hepatic fibrosis, a wound-healing response to chronic liver injury, is characterized by the activation of hepatic stellate cells (HSCs) and excessive deposition of extracellular matrix, representing a pivotal stage in the progression towards cirrhosis [66]. In this process, KCs are the key upstream cells that drive HSC activation. Upon liver injury, activated KCs release a plethora of pro-inflammatory and pro-fibrotic mediators (e.g., TNF-α, TGF-β, CCL2), which directly or indirectly drive the transdifferentiation of quiescent HSCs into a myofibroblast-like phenotype, thereby initiating the fibrogenic cascade [65]. The role of CRIg in this context is likely complex and multifaceted. As an immunoregulatory molecule, CRIg’s T-cell suppressive capacity may indirectly ameliorate fibrosis by mitigating immune-mediated hepatocyte damage. Furthermore, the efficient CRIg-mediated clearance of apoptotic hepatocyte debris could curtail the release of pro-inflammatory damage-associated molecular patterns (DAMPs), representing a potential anti-fibrotic mechanism [7,72,73]. However, emerging evidence suggests a seemingly profibrotic association. For instance, an abundance of CRIg-positive multinucleated giant cells (KC-derived syncytia) has been observed in cirrhotic patient tissues, and CRIg expression levels have been shown to correlate with disease severity in non-alcoholic steatohepatitis (NASH) models. Paradoxically, functional studies have revealed an intrinsic anti-fibrotic role for CRIg+ KCs. Li et al. demonstrated that Vsig4+ resident single-Kupffer cells improve hepatic inflammation and fibrosis in NASH [16]. Zhao et al. further showed that ALK1 signaling maintains KC homeostasis and prevents liver fibrosis, with KC depletion leading to enhanced fibrogenesis, highlighting the importance of the CRIg+ KC population in anti-fibrotic defense [23]. These findings imply that under conditions of persistent chronic inflammation, the observed accumulation of CRIg-positive KCs may undergo phenotypic transitions or represent a compensatory response, and the definitive role of CRIg in hepatic fibrosis likely depends on the specific stage of liver injury and prevailing microenvironmental cues. A recent comprehensive review by Liu et al. has summarized the biology of *VSIG4* and its implications for immune-mediated diseases, proposing that therapeutic *VSIG4* modulation may represent a viable strategy for managing fibrotic liver diseases [74]. Furthermore, emerging evidence supports this therapeutic potential by demonstrating that targeted regulation of the *VSIG4* pathway can effectively attenuate hepatic fibrotic progression.

### 5.3. CRIg and Hepatocellular Carcinoma (HCC)

Chronic liver inflammation and fibrosis create a pro-tumorigenic microenvironment that drives malignant transformation to HCC. Hepatic immune cells, including KCs and their derivative tumor-associated macrophages (TAMs), exhibit a pronounced duality in HCC pathogenesis: they can exert anti-tumor surveillance, yet are often “co-opted” by the tumor, polarizing towards an M2-like phenotype that fosters tumor growth, angiogenesis, and immune evasion [75].

In various malignancies, CRIg expression in TAMs is frequently associated with an immunosuppressive tumor microenvironment and correlates with poor patient prognosis [76]. If CRIg expression is maintained or upregulated in KC-derived TAMs, it may facilitate HCC progression via several mechanisms: (1) Enhanced immune evasion: CRIg could directly suppress the cytolytic activity of tumor-specific cytotoxic T lymphocytes (CTLs) through its potent co-inhibitory signaling. (2) Shaping a pro-tumor microenvironment: This may promote TAM polarization towards an M2-like state, leading to the secretion of factors that support tumor growth and angiogenesis (e.g., VEGF and IL-10). (3) Affecting antigen presentation: Phagocytosis of tumor-derived debris by CRIg-expressing TAMs could alter the initiation and quality of the anti-tumor immune response.

### 5.4. CRIg in Non-Alcoholic Fatty Liver Disease (NAFLD)

Metabolically dysregulated steatotic liver disease (MASLD), formerly known as NAFLD, and its progressive form, metabolic dysfunction-associated steatohepatitis (MASH/NASH), have emerged as the most common chronic liver diseases worldwide. The innate immune response contributes significantly to hepatic steatosis and disease progression. Recent studies have highlighted the critical protective role of CRIg-expressing Kupffer cells (KCs) in the pathogenesis of MASLD/NAFLD.

At the molecular level, the expression of CRIg (encoded by the *VSIG4* gene) is markedly downregulated in the fatty livers of NAFLD patients and obese mice. Li et al. demonstrated that *Vsig4* knockout accelerates high-fat diet (HFD)-induced metabolic dysfunction, promoting insulin resistance and lipid deposition. Mechanistically, the loss of CRIg exacerbates hepatic inflammation and fibrosis by unleashing the NF-κB signaling pathway, indicating that CRIg functions as a critical checkpoint to restrain inflammation during metabolic stress [15].

A key mechanism underlying this protective effect involves the clearance of harmful microbial products from the portal circulation. Luo et al. revealed that CRIg+ macrophages capture gut microbiota-derived extracellular vesicles (mEVs) from the bloodstream via a C3-dependent opsonization mechanism. This clearance function prevents the accumulation of microbial DNA in hepatocytes and hepatic stellate cells, thereby inhibiting the activation of the cGAS/STING pathway and subsequent fibrogenic inflammation [18].

Beyond diet-induced models, single-cell transcriptomic studies have provided insights into the role of CRIg+ macrophages in environmental pollutant-induced liver injury. Liu et al. analyzed the liver immune microenvironment in mice with fatty liver induced by microplastics. Their study identified CRIg+ macrophages as a liver-protecting subpopulation. Interestingly, microplastic exposure significantly increased the infiltration of CRIg+ macrophages while decreasing the angiogenic S100A6+ macrophage subpopulation, suggesting a compensatory protective response by KCs to maintain hepatic homeostasis under environmental stress [77].

Furthermore, bioinformatics analysis by Zhang et al. identified *VSIG4* as a top hub gene significantly enriched in immune response and macrophage regulation pathways in both NAFLD and heart failure [78]. This finding suggests that CRIg-mediated signaling may represent a shared molecular mechanism protecting against inflammation across different metabolic diseases.

## 6. Summary and Perspectives

Liver diseases represent a major global health burden, with diverse etiologies including viral hepatitis, metabolic disorders (NAFLD/NASH), alcohol consumption, and malignant transformation to HCC. Kupffer cells, as core cells of the liver innate immunity, are pivotal determinants of the host response to various liver insults. CRIg, a key complement receptor and immunoregulatory molecule prominently expressed on KCs, executes complex and context-dependent, often dichotomous functions in the context of liver diseases. While CRIg-mediated phagocytosis contributes to the clearance of opsonized particles, its potent immunosuppressive functions may concurrently promote T cell tolerance and contribute to liver diseases. The modulation of KC polarization and cytokine profiles by CRIg further influences the trajectory of hepatic inflammation and fibrosis.

However, the precise roles and dynamic changes in CRIg-mediated KCs throughout liver diseases, particularly during the transition to chronicity, fibrosis, and hepatocarcinogenesis, remain unclear. For example, how is CRIg expression and function modulated by variables such as gut microbiome composition, metabolic stress, inflammatory milieu, and host genetic factors? What is the nature of the crosstalk between CRIg-mediated phagocytic signaling and its T cell inhibitory pathways? How do KCs integrate these potentially conflicting signals to determine functional outcomes? Do CRIg-expressing KCs exhibit functional plasticity across different phases of liver disease, acting as either “drivers” of inflammatory damage or “guardians” of immune homeostasis?

Addressing these fundamental questions will not only advance our comprehensive understanding of the immunopathological mechanisms underlying liver diseases, but is also imperative for the rational design of novel therapeutic strategies. Targeting the CRIg axis may offer a promising avenue to reprogram the hepatic immune environment, modulate inflammation, prevent fibrosis progression, and ultimately improve outcomes across the spectrum of liver diseases.

## Data Availability

No new data were created or analyzed in this study. Data sharing is not applicable to this article.
